# Cost-effectiveness analysis of two-way texting for post-operative follow-up in Zimbabwe’s voluntary medical male circumcision program

**DOI:** 10.1371/journal.pone.0239915

**Published:** 2020-09-30

**Authors:** Joseph B. Babigumira, Scott Barnhart, Joanna M. Mendelsohn, Vernon Murenje, Mufuta Tshimanga, Christina Mauhy, Isaac Holeman, Sinokuthemba Xaba, Marrianne M. Holec, Batsirai Makunike-Chikwinya, Caryl Feldacker

**Affiliations:** 1 Department of Global Health, University of Washington, Seattle, WA, United States of America; 2 The Comparative Health Outcomes, Policy, and Economics (CHOICE) Institute, School of Pharmacy, University of Washington, Seattle, WA, United States of America; 3 International Training and Education Center for Health (I-TECH), Seattle, WA, United States of America; 4 Department of Medicine, University of Washington, Seattle, WA, United States of America; 5 International Training and Education Center for Health (I-TECH), Harare, Zimbabwe; 6 Zimbabwe Community Health Intervention Project (ZiCHIRE), Harare, Zimbabwe; 7 Medic Mobile, Nairobi, Kenya; 8 Ministry of Health and Child Care, Harare, Zimbabwe; University of Ghana College of Health Sciences, GHANA

## Abstract

**Objective:**

Although adverse events (AEs) following voluntary medical male circumcision (VMMC) are rare, their prompt ascertainment and management is a marker of quality care. The use of two-way text messaging (2wT) for client follow-up after VMMC reduces the need for clinic visits (standard of care (SoC)) without compromising safety. We compared the cost-effectiveness of 2wT to SoC for post-VMMC follow-up in two, high-volume, public VMMC sites in Zimbabwe.

**Materials and methods:**

We developed a decision-analytic (decision tree) model of post-VMMC client follow-up at two high-volume sites. We parameterized the model using data from both a randomized controlled study of 2wT vs. SoC and from the routine VMMC program. The perspective of analysis was the Zimbabwe government (payer). The time horizon covered the time from VMMC to wound healing. Costs included text messaging; both in-person and outreach follow-up; and AE management. Costs were estimated in 2018 U.S. dollars. The outcome of analysis was AE yield relative to the globally accepted safety standard of a 2% AE rate. We estimated the incremental cost per percentage increase in AE ascertainment and the incremental cost per additional AE identified. We conducted univariate and probabilistic sensitivity analyses.

**Results:**

2wT increased the costs due to text messaging by $4.42 but reduced clinic visit costs by $2.92 and outreach costs by $3.61 –a net savings of $2.10. 2wT also increased AE ascertainment by 50% (92% AE yield in 2wT compared to 42% AE yield in SoC). Therefore, 2wT dominated SoC in the incremental analysis: 2wT was less costly and more effective. Results were generally robust to univariate and probabilistic sensitivity analysis.

**Conclusions:**

2wT is cost-effective for post-VMMC follow-up in Zimbabwe. Countries in which VMMC is a high-priority HIV prevention intervention should consider this mHealth intervention to reduce overall cost per VMMC, increasing the likelihood of current and future VMMC program sustainability.

## Introduction

Voluntary medical male circumcision (VMMC) reduces the risk of female-to-male HIV-1 transmission by up to 60% [1–3]. As a result, nearly 19 million VMMCs were performed across 14 sub-Saharan African (SSA) countries from 2008 to 2017. VMMC is cost-effective for HIV prevention [4, 5] and is considered a priority for HIV prevention in SSA. In 2016, the UNAIDS set an annual target of 5 million VMMCs to reach 25 million men in SSA by 2021. However, many VMMC-priority countries face persistent healthcare resource constraints, characterized by low healthcare expenditures [6] personnel shortages [7] and an overdependence on donors for financing HIV prevention and treatment interventions [8]. These healthcare resource constraints threaten the quality and pace of VMMC scale-up [9, 10].

In VMMC programs, personnel shortages are a particularly difficult problem to address and assess as training and capacity building typically take a long time. Short-term monetary outlays, as are common with international donor-funded programs, don’t always include training beyond specific skillsets for singular program implementation, reducing long-term health personnel efficiency and impact. Program maintenance or sustainability is also threatened by continuous VMMC demand as successive cohorts of males come of age and traditional donor agencies plan to transfer VMMC programs to local Ministries and implementing partners. Resource shortages may also lead to cutting corners on program implementation, promoting program quantity over quality at scale [11].

Countries in which VMMC is of high priority have experienced a substantial increase in mobile health (mHealth) interventions. However, most mHealth interventions employ one-way (blast) messaging [12–14] which is less effective than bidirectional (two-way texting) communication that encourages interaction [15, 16]. In much of SSA, including Zimbabwe, mobile phone usage is widespread and presents an opportunity for mHealth innovation and deployment at scale. In the VMMC space, text message-based mHealth interventions have been applied with mixed success for demand creation [17], post-HIV testing demand creation [18], to deter early post-VMMC resumption of sexual activity [13, 19], and to encourage post-VMMC clinic visits [12].

In countries in which VMMC is of high priority, the intersection between increasing mHealth opportunities and healthcare resource constraints (human and financial) presents an opportunity for increased efficiency and cost reduction. While in-person follow-up after VMMC is critical for early detection of adverse events (AEs) [20], AEs are rare in routine program settings. Reported AE rates of large-scale VMMC programs are typically below the globally accepted AE rate of 2% [20–22]. Although the vast majority of VMMC recipients heal without complications [23], including those in Zimbabwe [24], in-person post-operative visits facilitate timely identification, management, and reporting of AEs. Recognizing both the importance of early AE ascertainment and the inefficiency of mandating in-clinic follow-up for the substantial majority of men who heal without complication, a randomized controlled trial (RCT) of post-VMMC follow-up using 2-way texting (2wT) was conducted in Zimbabwe [25]. In the RCT, VMMC clients were randomized to receive post-VMMC follow-up using 2wT or the standard of care (SoC). The 2wT arm required no in-person post-operative reviews while the SoC adhered to the national VMMC guidelines which required in-clinic follow-up visits on days 2, 7, and 42. Clinical outcomes from the RCT showed that 2wT-based follow-up was as safe as SoC: there was no significant difference in AE rates between groups [26]. However, 2wT ascertained and reported more AEs. As timely AE ascertainment is an indicator of VMMC program quality, 2wT-based follow-up appeared to improve patient care.

In this study, we assessed the cost-effectiveness of 2wT in Zimbabwe’s VMMC program. We hypothesized that 2wT would achieve gains in efficiency by reducing in-clinic post-VMMC follow-up visits. If 2wT proved more efficient in addition to being safe, swift replication of the intervention could better support VMMC sustainability. Due to the importance of safety and efficiency at scale, we sought to assess the tradeoff between identification of AEs (for quality VMMC management) and the costs of post-VMMC follow-up.

## Materials and methods

This study was approved by the Medical Research Council of Zimbabwe (MRCZ) and the University of Washington Institutional Review Board. All subjects received comprehensive information regarding their voluntary participation in the study and signed the written informed consent form prior to enrollment in the study. The trial was registered on ClinicalTrials.gov, trial NCT03119337, and activated on April 18, 2017. https://clinicaltrials.gov/ct2/show/NCT03119337.

We conducted a model-based cost-effectiveness analysis using primary data collected in the RCT of 2-way texting (2wT) for post-operative follow-up in Zimbabwe’s VMMC program [25]. The RCT was conducted in two high-volume VMMC clinics operated by the ZAZIC consortium, an implementing partner of the Zimbabwe Ministry of Health and Child Care’s (MoHCC) VMMC program. The ZAZIC VMMC approach adheres to MoHCC guidelines and was described previously [27]. The base case population was adult men (over 18 years) who underwent VMMC by dorsal slit. The study was conducted from the payer perspective, the Zimbabwe MoHCC. In the analysis, we considered only costs that would be incurred by the MoHCC. Costs to VMMC patients, such as transportation and time off work, were excluded. The study was conducted from a short-term time horizon corresponding to the time from VMMC to healing and as such, no discounting was necessary.

### Comparators

The comparators in the study were:

1. Standard of care (SoC)—post-VMMC wound care counseling; required in-person follow-up visits on post-surgery days 2, 7, and 42; instructions to seek care outside of scheduled visits on suspicion of AEs; and tracing with up to three phone calls followed by a home visit if clients did not return for the day 2 follow-up clinic visit.
2. Two-way texting (2wT)—enhanced post-VMMC counseling on use of 2wT, bandage removal, wound care, and AE recognition; automated daily texts from day 1–13 with requested responses; triaging of possible AEs via 2wT between patients and providers; and, return to clinic for suspicion and/or confirmation of AEs.

### Decision-analytic model

**Fig 1:**
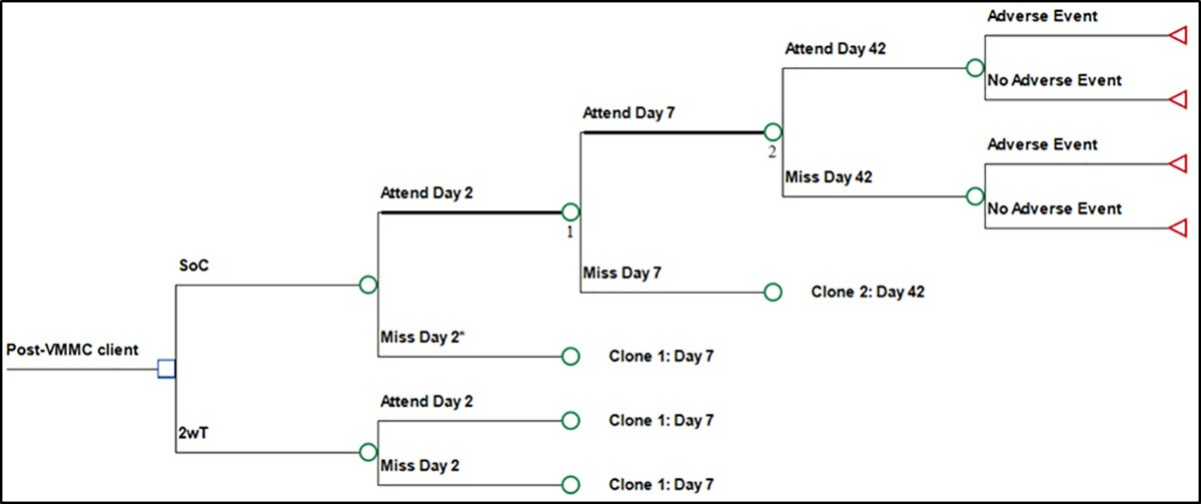
Decision analytic model (decision tree) of post-VMMC follow-up of men in Zimbabwe. *Leads to outreach. **Note the use of clones to complete the decision tree diagram after day 2 in the SOC and 2wT arms.

We developed a decision-analytic model (decision tree) to simulate the post-VMMC experience of members of the base case population. Fig 1 is a schematic of the model. After the VMMC procedure, clients in the SoC arm returned or did not return for follow-up on day two. Those who did not return on day two were followed up by phone calls and home visits. Clients returned or failed to return for follow-up on days 7 and 42. Those who did not return on days 7 and 42 were not followed further. In the 2wT arm, clients returned or failed to return on days 2, 7 and 42; 2wT clients who did not return were not followed up except through 2wT. Clients in both arms developed (or survived) VMMC-related AEs.

### Outcomes

#### Effectiveness of post-operative follow-up

The outcome was VMMC AE yield relative to the expected (maximum) rate of VMMC AEs in this population. The expected maximum rate of VMMC AEs in this population was assumed to be 2%, the commonly accepted safety standard [20–22]. Consequently, an AE yield less than 2% would be considered an underestimate of AEs while an AE yield of over 2% would be considered an overestimate of AEs. The percentage change in AE yield was calculated relative to 2% (i.e., percentage yield in SoC and 2wT divided by 2%) and expressed as a percentage. Data for effectiveness were obtained from the RCT, with reported AE rates as the base case estimates, and 95% confidence intervals were used in sensitivity analysis [25] Parameters for the outcome estimation are shown in Table 1.

**Table 1:** Outcome estimation parameters.

	Baseline	Range	Source
**Probabilities**			
Attend day 2 visit			
SOC	0.91	0.88–0.94	RCT*
2wT	0.05	0.03–0.08	RCT*
Attend day 7 visit			
SOC	0.52	0.47–0.57	RCT*
2wT	0.05	0.04–0.09	RCT*
Attend day 42 visit			
SOC	0.04	0.03–0.07	RCT*
2wT	0.03	0.02–0.06	RCT*
Adverse events			
SOC	0.0084	0.0028–0.0243	RCT*
2wT	0.0188	0.0086–0.0403	RCT*
**Cost parameters**			
Text messaging (2wT arm only)			
Commercial cost (per 250 texts)	$1.00	$0.50—$2.00	Econet [28]
Nurse wage (per month)	$907.00	$883.00—$931.00	MoH pay structure
FTEs dedicated to 2wT	0.17	0.08–0.25	Nurse time log
			RCT study logs
Mean number of texts per client	21.25	17.00–25.50	2wT call log
Duration of 2wT program (months)**	9	—	RCT study logs
Number of 2wT clients**	362	—	RCT study logs
Follow-up phone calls			
Commercial cost per call	$0.09	$0.05—$0.14	Econet [28]
			RCT study logs
FTEs dedicated to calls	0.022	0.018–0.026	Call logs
			RCT study logs
Mean number of calls per client	0.083	0.066–0.099	Call logs
			RCT study logs
In-clinic follow-up			
Clinic clerk wage (per month)	$700.00	$350.00—$1050.50	MoH pay structure
			RCT study logs
Clerk time (hours)	0.024	0.017–0.067	Time-motion
			RCT study logs
Nurse review time (hours)	0.083	0.017–2.033	Time-motion study
			RCT study logs
Hours worked per month	160	128–192	RCT study logs
Gloves	$0.20	$0.10—$0.30	MoHCC
Antiseptic	$0.50	$0.25—$0.75	
Gauze swabs	$0.20	$0.10—$0.30	
Bed liner	$0.20	$0.10—$0.30	
Distilled water	$0.10	$0.05—$0.15	
Alcohol rub	$0.30	$0.15—$0.45	
Outreach follow-up			
Mean distance to client home (km)	42.5	34.0–44.8	ZAZIC program logs
Liters of diesel (per km)	0.1	0.08–1.2	RCT study logs
Price of diesel (per liter)	$1.37	$0.69—$2.06	
Driver wage (per month)	$905.05	$452.53—$1,357	
Outreach time (hours)	1.125	0.900–1.350	
Phone-call during outreach	$0.09	$0.05—$0.14	
Lunch allowance	$10	$5—$15	
Adverse events			
Probability of bleeding	0.0097	0.0078–0.0117	RCT*
Probability of infection	0.0069	0.0055–0.0083	
Probability of swelling	0.0083	0.0067–0.0100	
Cost of surgery	$53.24	$26.62—$79.86	Assumption***
Cost of antibiotic	$5.00	$2.50—$10.00	Assumption
Cost of pain killer/ anti-inflammatory	$2.00	$1.00—$4.00	Assumption

*2wT RCT clinical outcome results published previously [26].

**Assumed no uncertainty.

***Cost of VMMC minus costs of circumcision kits [26].

#### Resource use and cost

We used a micro-costing approach for cost estimation. The parameter estimates, ranges used for sensitivity analysis, and data sources are presented in Table 1. We divided costs into the following categories: text messaging (2wT arm only), follow-up phone calls (as follow-up to 2wT), in-clinic post-VMMC follow-up, outreach post-VMMC follow-up (for missed day 2 appointments in SoC, including phone calls), and management of AEs.

The cost of text messaging for 2wT was calculated by summing the program costs of the text message service and the personnel cost of sending and responding to text messages. The cost of the text message service was calculated by multiplying the unit cost of a single text message by the mean number of texts sent per client in the trial. Because text messages are sold in bundles of 250, the cost of a single text message was estimated as a fraction of the bundle cost. The cost of 2wT-specific personnel was calculated using research logs to estimate the full time equivalents (FTE) spent by the nurse responsible for managing the 2wT system (including sending and responding to texts), and multiplying the FTE estimate by wage. Reports from research staff showed that the FTE required by the nurse to manage the texting was estimated at five to 15 minutes per hour, assuming an 8-hour work day and five working days a week, over the nine-month time period of the trial. The cost of follow-up phone calls made to 2wT clients was calculated by summing the costs of the phone service and the personnel costs of making calls. The cost of the phone service was calculated by multiplying the unit cost of a call by the mean number of calls per client in the trial as shown in call logs.

The cost of in-clinic follow-up was calculated by summing the personnel cost of follow-up and the costs of clinical supplies provided by the MoHCC VMMC program. The personnel cost of in-clinic follow-up was estimated by multiplying the time spent per client (by one clinic clerk and one VMMC nurse) by their wages. The use of one clinical clerk and one VMMC nurse reflects the pattern of routine post-VMMC follow-up in the MoHCC VMMC program. The cost of supplies included the components of a MoHCC wound dressing bundle of gloves, antiseptic, alcohol rub, gauze, bed liner, and distilled water.

The cost of follow-up for missed day two visits in SoC was calculated by summing the personnel costs of outreach, transportation costs, costs of phone calls (made during attempts to reach client’s homes), and costs of clinical supplies obtained from both RCT records and VMMC program logs. The costs of personnel were calculated based on the common practice of using one driver and one nurse for home visits and multiplying their wages by the time spent in this outreach activity. The nurse and driver also received a lunch allowance per outreach which was also included. The costs of phone calls during outreach assumed an average of one phone call per client home visit. The cost of clinical supplies included the components of a wound dressing bundle as described above. The costs of outreach transportation were calculated by multiplying the cost of fuel per unit distance by the mean distance of follow-up, both obtained from program data.

The costs of AE management were calculated by multiplying the probability of occurrence of the AE type from the trial (bleeding, infection, and swelling) [25] by the costs of managing the different AE types (minor surgery, antibiotics, and analgesics/antipyretics). The cost of minor surgery was assumed to equal to the cost of dorsal slit VMMC minus the cost of circumcision kits. All costs were estimated in 2018 US dollars ($US).

### Analysis

The base case analysis compared: 1) the mean cost and adverse event yield of 2wT to SoC; and 2) the incremental cost-effectiveness ratio (ICER)—the cost per percentage increase in AE yield; and 3) cost per additional AE identified (relative to the expected number)—comparing 2wT to SoC. We conducted univariate sensitivity analyses to assess the most influential parameters. All parameters were varied across plausible ranges: we used 95% confidence intervals (CIs) of empirical estimates and, where 95% CIs were unavailable, we employed ranges of +/- 20% for probabilities and +/- 50% for costs [29–31]. We also conducted probabilistic sensitivity analyses using Monte Carlo simulation to assess overall model parameter uncertainty and to further test the robustness of the results. We used the baseline values as means and estimated standard errors assuming ranges were equivalent to 95% CIs (four times the standard error). We assumed beta distributions for probabilities, gamma distributions for costs, and normal distributions for other variables.

## Results

### Base-case analysis

The base case results are shown in Table 2. 2wT-specific activities increased the mean costs of post-VMMC follow-up by $4.42 overall: $0.09 in text service costs, $3.83 in text-related personnel costs, $0.01 in phone call costs and $0.50 in call-related personnel costs. However, the 2wT intervention also reduced costs: clinic visits decreased by $2.92, outreach visits by $3.61, and AE management by $0.002. Therefore, the net impact was that 2wT reduced the mean costs of VMMC follow-up by $2.10. The 2wT intervention also increased AE ascertainment (as compared to the expected AE yield of 2%) by 50% (92% AE yield in 2wT compared to 42% AE yield in SoC). Therefore, 2wT dominated SoC in the incremental analysis: 2wT was less costly and more effective.

**Table 2:** Base case results.

	SoC	2wT	2wT vs. SoC
**Costs (of follow-up) per client**			
2wT			
Text (service)	—	$0.085	$0.085
Text (personnel)	—	$3.833	$3.833
Call back (service)	—	$0.007	$0.007
Call back (personnel)	—	$0.496	$0.496
Total costs of 2wT	$0.00	$4.42	$4.42
Clinic visits	$3.20	$0.28	– $2.92
Outreach visits	$3.61	—	– $3.61
Adverse event management	$0.003	$0.001	– $0.002
Total costs	$6.82	$4.72	– $2.10
**Outcomes**			
AE yield (vs. expected AE rate)*	42.00%	92.00%	50.00%
**Analysis**			
Cost per % increase in AE yield (vs. expected rate)	—	—	2wT dominant**
Cost per additional AE identified (vs. expected #)	—	—	2wT dominant**

*Calculated as AE rate divided by expected rate (2%) for both SoC and 2wT, separately, and expressed as a percentage.

**Less costly and more effective.

### Sensitivity analysis

**Fig 2:**
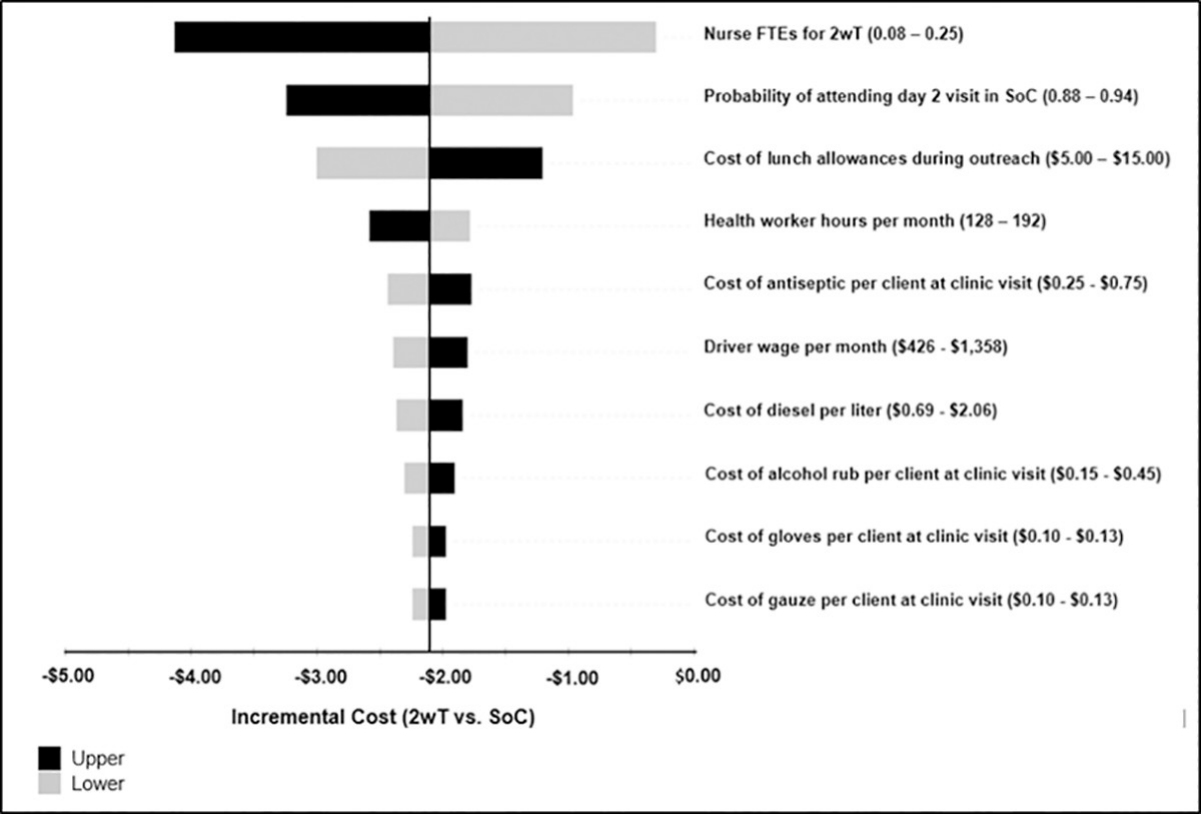
One-way sensitivity analysis on incremental cost comparing 2wT to SoC. The top ten influential variables are shown. FTE–full time equivalent. 2wT–two-way texting. SoC—standard of care.

In univariate sensitivity analysis (Fig 2), the reduction in mean cost of post-VMMC follow-up in 2wT as compared to SoC was most sensitive to the FTE of nurses providing the 2wT service, the probability of attending (or missing) the day two visit in the SoC, and the lunch allowance provided to outreach personnel. This result was generally robust to univariate sensitivity analysis as the highest estimate of 2wT-related FTE was associated with a reduction of $0.25 compared to SoC.

**Fig 3:**
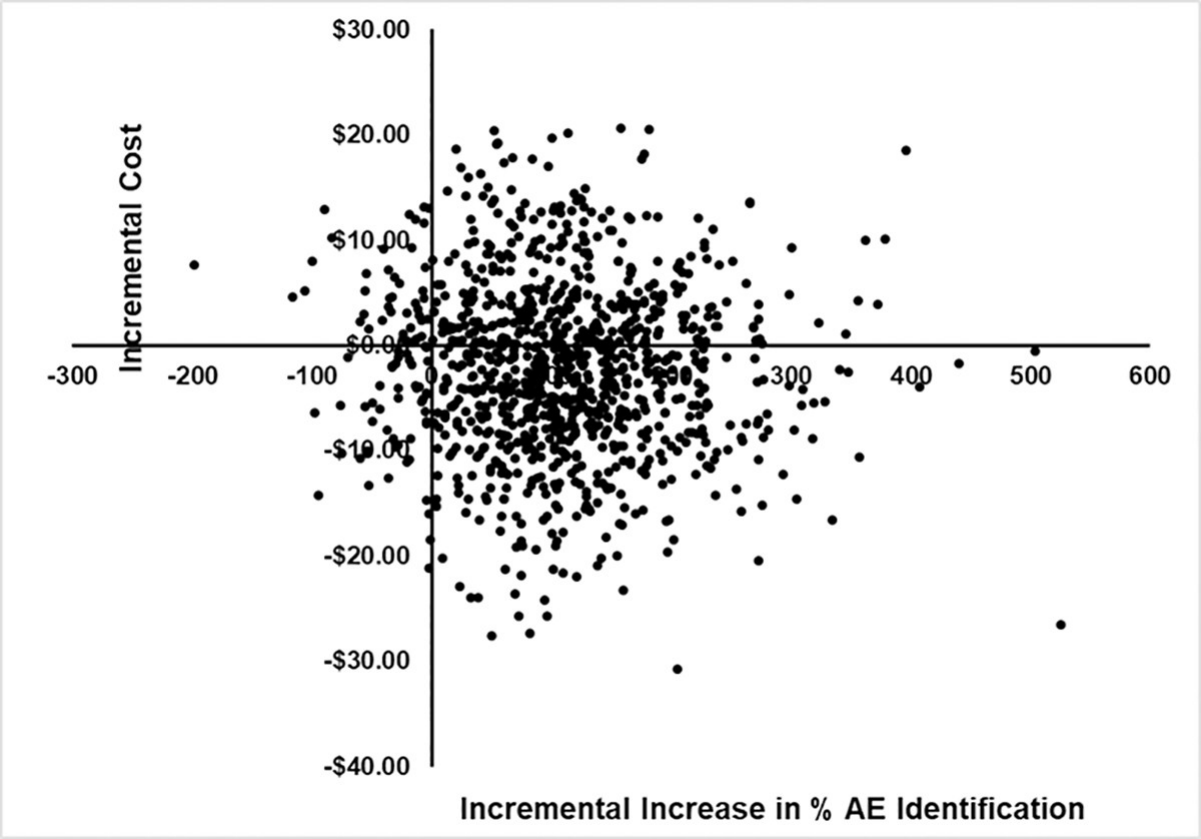
Incremental cost-effectiveness scatterplot of comparison of 2wT vs. SoC. AE–Adverse event.

The results of the probabilistic sensitivity analysis are shown in an incremental cost-effectiveness (ICE) scatterplot (Fig 3). Of the 1,000 Monte Carlo simulations, 53% of the cost-effectiveness pairs were in the “south-east” quadrant (increased effectiveness and reduced cost) of the cost-effectiveness plane, which is also the quadrant of the base case results. Of the 1,000 Monte Carlo simulations, 39% of the cost-effectiveness pairs were in the “north-east” quadrant (increased effectiveness and increased cost) of the cost-effectiveness plane. Much smaller proportions of the cost-effectiveness pairs from the 1,000 Monte Carlo simulations were in the “north-west” quadrant (3%) and the south-west quadrant (5%) of the cost-effectiveness plane.

## Discussion

2wT for VMMC follow-up appeared to increase AE ascertainment at reduced cost as compared to SoC. Although VMMC, itself, is cost-effective for HIV prevention, sustainability of VMMC programs will depend on innovations for cost-containment as donor funding expires and programs revert to Ministry funding in VMMC-priority countries. In this cost-effectiveness analysis of an mHealth intervention, 2wT was less costly and more effective in identifying AEs with dramatically reduced healthcare worker workload as compared to the SoC with in-person visits. VMMC programs in high-priority countries should consider this 2wT mHealth interventions to reduce the overall costs per VMMC while maintaining VMMC quality. The adoption of 2wT could contribute positively to long-term sustainability of VMMC programs.

The results of the probabilistic sensitivity analyses (Fig 3) showed that there was more uncertainty as to the impact of 2wT to reduce cost (as shown by the substantial distribution of cost-effectiveness pairs in the vertical (cost) plane of the ICE scatter plot) compared to its impact to increase effectiveness (as shown by the limited distribution of cost-effectiveness pairs in the horizontal (effectiveness) plane of the ICE scatter plot). As shown in Fig 3, there was a very small proportion of cost-effectiveness pairs in the north-west and south-west quadrants of the cost-effectiveness plans, suggesting a great deal of certainty as to the impact of 2wT to increase effectiveness. This suggests that there is more room for further innovation in cost-containment which may be easier achieved than innovation in effectiveness, in this case identifying adverse events. Such innovation to reduce costs, through for instance gains in efficiency in administering the 2wT program, will hasten the achievement of long-term sustainability of VMMC programs.

We used the payer perspective to assess the cost-effectiveness of 2wT. In Zimbabwe, the payer is the government, the MoHCC, which would likely bear the future costs of sustaining the VMMC program. However, broader potential benefits of 2wT should not be discounted. From a societal perspective, 2wT is likely to be even more economically attractive given that it would save client costs associated with in-person post-VMMC visits (e.g. transportation and missed wages). 2wT would also be expected to increase demand for VMMC by reducing the financial disincentives and perceived inconvenience of routine VMMC post-operative visits. For providers, less time on in-person follow-up reviews could free them to perform more VMMCs or other clinic duties.

From a bilateral and multilateral donor perspective, principally that of the major VMMC donor, the United States’ President's Emergency Plan for AIDS Relief (PEPFAR), the cost savings per VMMC using 2wT for follow-up is approximately $2.10. This amount is substantial if applied to the nearly 19 million VMMCs performed in the last 10 years. Additionally, the study showed a cost savings of $2.92 on in-person clinic visits. Applying this across the health sector, where relevant, would suggest a substantial potential reduction in the cost of VMMC in Zimbabwe and elsewhere. Furthermore, the internal ZAZIC program expenditure analysis conducted to comply with PEPFAR reporting requirements, estimated that from a programmatic perspective, the cost of service delivery within the overall ZAZIC program is 66% of the total cost of a VMMC–with 12% spent on demand creation and an additional 22% spent on management [32]. Therefore, a $2.10 reduction in follow-up costs could be shifted to other areas of service delivery and potentially allow for more VMMCs.

We may have underestimated the potential positive impact of 2wT on VMMC program cost savings. 2wT was implemented as a RCT in a routine VMMC setting. Therefore, the study does not provide a complete picture of the cost saving impact of 2wT. For instance, the 2wT nurse responsible for client communication was also part of other trial activities and provided VMMC services for a limited number of patients. It is possible that dedicated nurses for 2wT in high-volume sites or increasing numbers of patients enrolled in 2wT may have led to even greater cost savings.

There are several limitations of this analysis and its interpretation. First 2wT savings may differ in other contexts. Although 2wT reduces costs as compared to standard in-clinic follow-up in Zimbabwe, the amount of savings would depend on the specific staffing structure in other contexts. In settings with different human resources or implementation models, cost savings and the impact of 2wT on personnel distribution and use could differ. Secondly, we selected quality of care as the outcome—cost per AE yield. Although this is a critical indicator of patient safety, it does not immediately lend itself to comparison across interventions in the health sector. However, the 85% clinic workload reduction observed between the 2wT and the SoC should be a convincing measure of the impact of 2wT to reduce health resources utilization, helping demonstrate the potential economic advantages of 2wT in the broader healthcare system context. Finally, we did not include the costs of 2wT design, development, and maintenance in our analysis from the payer perspective, and we did not factor in the standard computers and Android phones for system operation. While we assumed that the marginal cost of inheriting an existing system by a government would be minimal on a per VMMC client basis, there may be significant periodic costs of upgrades and system maintenance. At scale, however, the capital and operating costs would be distributed across all circumcisions and would represent conservatively, at most, $.25 per VMMC by our estimates.

## Conclusion

This economic evaluation of a novel application of mHealth found that 2wT-based VMMC follow-up reduced provider workload while maintaining quality of care. By dramatically decreasing unnecessary visits, 2wT saved costs, increasing the potential for program sustainability. We conclude that in the ongoing context of extreme resource constraints in high-priority countries, 2wT is a cost-effective method to improve AE ascertainment and maintain the quality of VMMC care. Further expansion and testing of 2wT for VMMC follow-up is warranted.
